# Supplementary material for: Listening to Their Nights: Sleep Disruptions in Captive Housed Chimpanzees Affect Their Daytime Behavior
Source: Animals (Basel). 2023 Feb 16;13(4):696. doi: 10.3390/ani13040696 (PMC9952389; doi:10.3390/ani13040696)
Supplement: Supplementary file 1 [file animals-13-00696-s001.zip › animals-2192854-supplementary.pdf]

# Supplementary Material

# Listening to their nights: Sleep disruptions in captive housed chimpanzees affect their daytime behavior

Pablo R. Ayuso, Olga Feliu, David Riba and Dietmar Crailsheim

**Table S1**. Characteristics of the study population.

| Name | ID | Sex | Age category | Group |
|---|---|---|---|---|
| Africa | AFR | F | Adult | Mutamba |
| Bongo | BON | M | Adult | Mutamba |
| Juanito | JUA | M | Adult | Mutamba |
| Marco | MAR | M | Senior | Mutamba |
| Waty | WAT | F | Adult | Mutamba |
| Bea | BEA | F | Senior | Bilinga |
| Cheeta | CHE | F | Senior | Bilinga |
| Coco | COC | F | Adult | Bilinga |
| Nico | NIC | M | Adult | Bilinga |
| Tico | TIC | M | Senior | Bilinga |
| Tom | TOM | M | Senior | Bilinga |
| Victor | VIC | M | Senior | Bilinga |

Abbreviations: F = Female; M = Male

**Table S2. This table show the ethogram we used to record daytime behavioral data.**

| Type of Behavior | Name of behavior | Definition |
|---|---|---|
| Solitary | Abnormal | This includes: (a) Atypical Behaviors, i.e. behaviors not typically seen in wild populations; (b) behaviors exhibited in an excessive manner or as an inadequate, non-adaptive response to a specific situations. SELF SCRATCH: invariable, rhythmic and repetitive movement of the hand fingers or with an object (e.g. stick) rubbing some part of its own body. SELF POKE: insert or press fingers or objects (e.g. sticks) repeatedly or continuously in a part of the body (only mark when actively manipulating or maintaining contact). SELF CLAP: hitting (soft or hard) some part of the body with hands or feet rhythmically and repetitively. ROCKING: Repetitive and invariable swinging of the body while sitting (they can hug or grab objects at the same time, e.g. pile of straw). OVER GROOMING: excessive self-cleaning behavior of the skin / coat with hands or mouth, which may include pulling and ingesting hair. The behavior can also be focused on wounds or damage to the skin, deteriorating its condition. They usually do it on the same areas of the body with a visible lack of hair. COPROPHAGY: handling and/or ingestion of feces (both own and others). OTHER ABNORMAL: other abnormal or stereotyped behaviors not described above. The majority can be identified due to its rhythmically, repetitiveness and/or excessive frequency. |
| Solitary | Locomotion | Displacement from a point A to a point B on a vertical or horizontal surface, while not doing any other behavior from the catalogue. |
| Solitary | Alimentation | All behaviors related to food. FOOD: ingest or handle solid or liquid food in a stationary position. FOODING: active search and/or transport of food (the animal keeps its eyes focused on possible food sources). It can include punctual food intake without interrupting gathering food. FOOD EXTRACTION: The chimpanzee is working an enrichment device/item (provided by care givers) which contains food items. This may include longer periods of manipulation in order to bring out the food items which are occasionally ingested. The chimpanzee might use feet, hands, mouth or tools. |
| Solitary | Manipulation | Inspecting elements of the environment or enrichment that are not food with the upper or lower extremities. |
| Solitary | Inactivity | RESTING: the individual remains stationary without actively interacting with himself, other individuals, nor does VIGILANCE or RELAXED behavior. VIGILANCE BEHAVIOR: described as the fixed gazing of something concrete or scanning the distant environment in a fixed, immobile position, either seated or bipedal standing. It does not include looking at parts of your own body, objects in close proximity, or other individuals within a 5m radius. It includes looking at distant group members, other groups, and the surrounding area. RELAXED RESTING: dozing or sleeping in an upward-facing or lateral position with either elongated or bent limbs (without hugging oneself). The individual does not show any indication of being focused on any other individual or their general environment. The individual is not defined as RELAXED RESTING if it is goes into a fetal position (may include hugging limbs and/or patting oneself with fingers on various parts of the body), nor any other behavior categorized as abnormal, such as: self-poking or self-clasping, etc. |

| | | |
|---|---|---|
| Solitary | Self Directed | Behaviors directed towards the individual, such as self-cleaning (auto grooming), masturbation, scratching, scrubbing, body inspection, etc. |
| Solitary | Other Individual | Individual behaviors that are not better defined by any other category of the "Solitary Behavior" group. Does include behaviors such as individual tension or excretion. SOLITARY BLURRED: mark only when the individual can be located performing an individual behavior but the behavior the individual performs is not identified due to lack of vision. |
| Solitary | Solitary Play | All Solitary Play behaviors have to be either accompanied by either laughs or a "play face". (In the absence of laughter or a play face this would be recorded as "Manipulation"). For all categories, be careful not to include abnormal behaviors such as self-clasping, self-patting, pacing, self-poking, body rocking, or any other abnormal or stereotypical behaviors. LOCOMOTOR SOLITARY PLAY: An active game played by a single individual that includes swinging, hanging, jumping, pivoting, sliding, jumping and moving quickly without any indication of external motivation (social or nutritional, for example). STATIONARY SOLITARY PLAY: the individual plays silently with the hands, the fingers and toes, or other parts of one's own body. OBJECT SOLITARY PLAY: the individual plays individually while carrying or using one or several objects. |
| Other | Not visible | The individual cannot be located or is out of the view of the observer. INTERIORS: mark when the individual is in the bedrooms while having access to the observed group exteriors. |
| Other | Human Positive | Interaction, or looking for interaction, with humans of a neutral type, or without showing clear signs of antagonism. HUMAN AFFILIATIVE INTERACTION: search, action, or mutual influence with or without physical contact between animal and human of non-agonistic type. It can be initiated by animals or by humans. OBSERVING HUMAN CLOSE UP: the individual remains less than 1.5 meters from the fence observing humans in close proximity. FOLLOW HUMAN: locomotion in parallel to the fence following the path that people make around the enclosure. |
| Other | Human Negative | Agonistic-like interaction or looking for it towards humans. HUMAN AGGRESSION: chimpanzees show threatening behavior and/or perform an agonistic display directed at humans in close proximity. It may include attempts to come into physical contact, throw items, or spit. HUMAN SUBMISSIVE: Chimpanzees are agitated with a scared face can include behaviors such as asking for support, crying or yelling directed at a human. |
| Social | Grooming | Body-cleansing behavior (grooming) from one individual to another (includes mutual grooming), performed with the upper extremities or with the mouth. |
| Social | Agonistic Dominance | Behaviors relating to the agonistic/display threat, direct aggression, displacement and resource appropiation (including object and social resources) It can be accompanied by vocalizations. |
| Social | Agonistic Submission | The chimpanzee shows signs of fear directed at another individual in an aggressive context. It may include approaching and presenting vulnerable body parts in an attempt to appease or attempt to avoid or flee from an individual displaying aggressive behavior. It can include actions such as genital presenting, extending limbs, hand to mouth, finger to mouth, running away, avoiding, and is typically accompanied by a scared face and vocalizations such as yelling, screaming, crying or pant-grunting. |
| Social | Other Agonistic | Other aggression-related behaviors that cannot be defined by the Agonistic Dominance or Agonistic Submission categories. AGONISTIC BLURRED: the individual can be located performing or receiving agonistic social behavior, but the behavior the individual performs or receives is not identified due to lack of vision. |

| | | |
|---|---|---|
| Social | Social Play | Playful behavior between two or more individuals punctually accompanied by playful indicators (eg, play face, laugh, gallop, cartwheels and pirouettes). Tag, catch and grab limbs or objects, tickles, muzzling, rough-and-tumble. These interactions do not require permanent physical contact. |
| Social | Other Affiliative | Other affiliative-like behaviors that cannot be defined by the "Social Play" or "Grooming" categories. Any of the following 3 subcategories are marked if they fit the following definitions: FOLLOW: Synchronized movement following the trajectory of another individual locomotion following the trajectory of another individual, moving beside or behind (always mark mutual). EMBRACE: An individual hugs or tries to hug another with one or both arms. FEED TOGETHER Two individuals eat from the same food source or forage in close proximity allowing the collection of food within their reach by another individual, sharing the food in a passive way (always marking it mutual). AFFILIATIVE BLURRED: the individual can be located performing or receiving an affiliative social behavior but the behavior he / she performs or receives is not identified due to lack of vision. |
| Social | Socio Sexual | Sexual interaction, or search for sexual interaction, between two individuals that includes behaviors such as: copulation, attempted copulation, genital presentation, and other behaviors directed towards the genitals of another individual. |
| Distance | Social proximity | Sharing a space during inactivity, or associated with other individual behaviors, while the distance between individuals is less than the length of the upper extremity. (Includes physical contact). Social Proximity is only recorded between animals that do not interact socially. |

**Table S3. Minimum, maximum, average and standard deviation values of the sample used in the analysis for the different variables recorded.**

| | Minimum | Maximum | Average | Standard deviation |
|---|---|---|---|---|
| **Average Tª (ºC)** | 16.6 | 28.6 | 22.0 | 5.5 |
| **Max Humidity (%)** | 49 | 99 | 78.1 | 9.2 |
| **Min Humidity (%)** | 27 | 91 | 58.7 | 13.7 |
| **Nº events** | 1 | 6 | 2.2 | 1.1 |
| **Max Events (seconds)** | 286 | 4771 | 1065.4 | 452.2 |

**Table S4. Categories of variables used for data analysis: number of events (Nº Events) and duration of the longest event (Max Duration).**

| Variable | Category | Range |
|---|---|---|
| **Nº events** | one | 1 |
| | two | 2 |
| | three or more | 3 - 6 |
| **Max Events (seconds)*** | short | < 870 |
| | medium | 870 – 1150 |
| | long | > 1150 |

* The cut-off values between categories have been assigned to achieve an equal distribution of the data in the three categories.

**Table S5.** Results of Kendall's rank correlation test for all measured abiotic factor variables (adjusted p values with Holm-Bonferroni method).

| Night disruption | Abiotic factor | tau | Z | p |
|---|---|---|---|---|
| **Nº events** | **Tª average** | 0.2717223 | 5.553 | 2.808e-08 ** |
| | **Tª min** | 0.3015013 | 6.1065 | 1.018e-09 ** |
| | **Tª max** | 0.23958 | 4.8745 | 1.091e-06 ** |
| | **H average** | -0.05452151 | -1.1154 | 0.2647 |
| | **H min** | -0.002396573 | -0.048865 | 0.961 |
| | **H max** | -0.1376107 | -2.7921 | 0.005237 ** |
| **Max Events** | **Tª average** | 0.1169002 | 2.7095 | 0.006739 ** |
| | **Tª min** | 0.1236477 | 2.8403 | 0.004506 ** |
| | **Tª max** | 0.1137081 | 2.6239 | 0.008694 ** |
| | **H average** | -0.01373435 | -0.31867 | 0.75 |
| | **H min** | 0.000268284 | 0.006204 | 0.995 |
| | **H max** | -0.04058961 | -0.93404 | 0.3503 |

Signif. codes: p <0.05 ' * ' / p<0.01 ' ** '

**Table S6. Linear mixed models based on ANOVA Type III Analysis.**

| | | | Anova Type III Analysis of Variance Table with Satterthwaite's method | | | | | | | | | | | | | | | |
|---|---|---|---|---|---|---|---|---|---|---|---|---|---|---|---|---|---|---|
| | | | Nº Events | | | | Max Event | | | | Sex | | | | Age Category | | | |
| **Model** | **ΔAIC** | **p** | **SumSq** | **Mean Sq** | **Fvalue** | **p** | **SumSq** | **Mean Sq** | **Fvalue** | **p** | **SumSq** | **Mean Sq** | **Fvalue** | **p** | **SumSq** | **Mean Sq** | **Fvalue** | **p** |
| **Inactivity** | 57.5 | 5.221e-13 ** | 0.163633 | 0.081816 | 26.3382 | 8.258e-10 ** | 0.122320 | 0.061160 | 19.6885 | 7.203e-08 ** | 0.003445 | 0.003445 | 1.1090 | 0.31296 | 0.019007 | 0.019007 | 6.1186 | 0.02924 * |
| **Abn - Self** | 16.15 | 8.798e-05 ** | 0.0013494 | 0.0006747 | 0.6774 | 0.5104 | 0.0312790 | 0.0156395 | 15.7027 | 1.306e-06 ** | 0.0000074 | 0.0000074 | 0.0075 | 0.9326 | 0.0000722 | 0.0000722 | 0.0725 | 0.7923 |
| **Aff - Prox** | 44 | 2.911e-10** | 0.111800 | 0.055900 | 15.6418 | 1.342e-06 ** | 0.131204 | 0.065602 | 18.3566 | 1.817e-07 ** | 0.013621 | 0.013621 | 3.8114 | 0.07459 | 0.001056 | 0.001056 | 0.2954 | 0.59674 |

Signif. codes: p <0.05 ‘ * ’ / p<0.01 ‘ ** ’

**Table S7.** Post hoc test results of the linear mixed models. Multiple comparison of means with Tukey contrasts (adjusted p values with Holm-Bonferroni method).

| Model | Fixed Factor | | | Estimate | Std. Error | Z value | p value |
|---|---|---|---|---|---|---|---|
| **Inactivity** | **Nº Events** | one | two | 0.06250 | 0.01324 | 4.720 | 4.73e-06 ** |
| | | one | three | 0.09530 | 0.01335 | 7.141 | 2.78e-12 ** |
| | | two | three | 0.03280 | 0.01324 | 2.476 | 0.0133 * |
| | **Max Event** | short | medium | -0.02817 | 0.01314 | -2.144 | 0.032 * |
| | | short | long | 0.05446 | 0.01335 | 4.079 | 9.03e-05 ** |
| | | medium | long | 0.08263 | 0.01335 | 6.189 | 1.81e-09 ** |
| | **Age Category** | adult | senior | 0.10030 | 0.04055 | 2.474 | 0.0134 * |
| **Abn - Self** | **Max Event** | short | medium | 0.001556 | 0.007439 | 0.209 | 0.834 |
| | | short | long | 0.037632 | 0.007559 | 4.978 | 1.92e-06 ** |
| | | medium | long | 0.036076 | 0.007559 | 4.772 | 3.64e-06 ** |
| **Aff - Prox** | **Nº Events** | one | two | 0.05980 | 0.01420 | 4.210 | 5.11e-05 ** |
| | | one | three | 0.07543 | 0.01420 | 5.310 | 3.28e-07 ** |
| | | two | three | 0.01563 | 0.01409 | 1.109 | 0.267 |
| | **Max Event** | short | medium | -0.01016 | 0.01409 | -0.721 | 0.471 |
| | | short | long | 0.06912 | 0.01420 | 4.866 | 2.27e-06 ** |
| | | medium | long | 0.07928 | 0.01420 | 5.581 | 7.15e-08 ** |

**Table S8.** Post hoc test (emmeans function) results of the LMM regarding Inactivity, including fixed factor interaction between Max Duration and Nº Events, based on pairwise comparison of means. Results are averaged over the levels of Sex and Age Category with Tukey contrast for comparing a family of 9 estimates (adjusted p-values with Bonferroni method)

| Interaction Max duration : Nº events | | estimate | SE | df | t ratio | p value |
|---|---|---|---|---|---|---|
| **short + one** | medium + one | 0.0282 | 0.0134 | 98.2 | 2.0980 | 0.4808 |
| **short + one** | long + one | -0.0545 | 0.0136 | 98.2 | -3.9910 | 0.0039** |
| **short + one** | short + two | -0.0625 | 0.0135 | 98.2 | -4.6180 | 0.0004*** |
| **short + one** | medium + two | -0.0343 | 0.0191 | 98.2 | -1.8010 | 0.6814 |
| **short + one** | long + two | -0.1170 | 0.0191 | 98.2 | -6.1350 | <.0001*** |
| **short + one** | short + three or more | -0.0953 | 0.0136 | 98.2 | -6.9860 | <.0001*** |
| **short + one** | medium + three or more | -0.0671 | 0.0191 | 98.2 | -3.5070 | 0.0189* |
| **short + one** | long + three or more | -0.1498 | 0.0193 | 98.2 | -7.7620 | <.0001*** |
| **medium + one** | long + one | -0.0826 | 0.0136 | 98.2 | -6.0550 | <.0001*** |
| **medium + one** | short + two | -0.0907 | 0.0191 | 98.2 | -4.7560 | 0.0002*** |
| **medium + one** | medium + two | -0.0625 | 0.0135 | 98.2 | -4.6180 | 0.0004*** |
| **medium + one** | long + two | -0.1451 | 0.0191 | 98.2 | -7.6130 | <.0001*** |
| **medium + one** | short + three or more | -0.1235 | 0.0191 | 98.2 | -6.4510 | <.0001*** |
| **medium + one** | medium + three or more | -0.0953 | 0.0136 | 98.2 | -6.9860 | <.0001*** |
| **medium + one** | long + three or more | -0.1779 | 0.0193 | 98.2 | -9.2220 | <.0001*** |
| **long + one** | short + two | -0.0080 | 0.0194 | 98.2 | -0.4150 | 1.0000 |
| **long + one** | medium + two | 0.0201 | 0.0194 | 98.2 | 1.0390 | 0.9810 |
| **long + one** | long + two | -0.0625 | 0.0135 | 98.2 | -4.6180 | 0.0004*** |
| **long + one** | short + three or more | -0.0408 | 0.0193 | 98.2 | -2.1170 | 0.4684 |
| **long + one** | medium + three or more | -0.0127 | 0.0193 | 98.2 | -0.6570 | 0.9992 |
| **long + one** | long + three or more | -0.0953 | 0.0136 | 98.2 | -6.9860 | <.0001*** |
| **short + two** | medium + two | 0.0282 | 0.0134 | 98.2 | 2.0980 | 0.4808 |
| **short + two** | long + two | -0.0545 | 0.0136 | 98.2 | -3.9910 | 0.0039** |
| **short + two** | short + three or more | -0.0328 | 0.0135 | 98.2 | -2.4230 | 0.2835 |
| **short + two** | medium + three or more | -0.0046 | 0.0191 | 98.2 | -0.2430 | 1.0000 |

| | | | | | | |
|---|---|---|---|---|---|---|
| **short + two** | long + three or more | -0.0873 | 0.0194 | 98.2 | -4.5040 | 0.0006*** |
| **medium + two** | long + two | -0.0826 | 0.0136 | 98.2 | -6.0550 | <.0001*** |
| **medium + two** | short + three or more | -0.0610 | 0.0191 | 98.2 | -3.1980 | 0.0466 |
| **medium + two** | medium + three or more | -0.0328 | 0.0135 | 98.2 | -2.4230 | 0.2835 |
| **medium + two** | long + three or more | -0.1154 | 0.0194 | 98.2 | -5.9580 | <.0001*** |
| **long + two** | short + three or more | 0.0217 | 0.0191 | 98.2 | 1.1360 | 0.9671 |
| **long + two** | medium + three or more | 0.0498 | 0.0191 | 98.2 | 2.6140 | 0.1944 |
| **long + two** | long + three or more | -0.0328 | 0.0135 | 98.2 | -2.4230 | 0.2835 |
| **short + three or more** | medium + three or more | 0.0282 | 0.0134 | 98.2 | 2.0980 | 0.4808 |
| **short + three or more** | long + three or more | -0.0545 | 0.0136 | 98.2 | -3.9910 | 0.0039** |
| **medium + three or more** | long + three or more | -0.0826 | 0.0136 | 98.2 | -6.0550 | <.0001*** |

Signif. codes: p <0.05 ‘ * ’ / p<0.01 ‘ ** ’ p<0.001 ‘ *** ’

Table S9: Post hoc test (emmeans function) results of the LMM regarding Inactivity, including fixed factor interaction between Age Category and Nº Events, based on pairwise comparison of means. Results are averaged over the levels of Sex and Max Duration with Tukey contrast for comparing a family of 6 estimates (adjusted p-values with Bonferroni method)

| **Interaction: Age Category : Nº events** | | **estimate** | **SE** | **df** | **t ratio** | **p value** |
|---|---|---|---|---|---|---|
| **adult + one** | senior + one | -0.1003 | 0.0764 | 9.55 | -1.3140 | 0.7721 |
| **adult + one** | adult + two | -0.0625 | 0.0135 | 98.21 | -4.6180 | 0.0002*** |
| **adult + one** | senior + two | -0.1628 | 0.0775 | 11.62 | -2.1000 | 0.3498 |
| **adult + one** | adult + three or more | -0.0953 | 0.0136 | 98.24 | -6.9860 | <.0001*** |
| **adult + one** | senior + three or more | -0.1956 | 0.0775 | 11.72 | -2.5230 | 0.1934 |
| **senior + one** | adult + two | 0.0378 | 0.0776 | 11.76 | 0.4870 | 0.9957 |
| **senior + one** | senior + two | -0.0625 | 0.0135 | 98.21 | -4.6180 | 0.0002*** |
| **senior + one** | adult + three or more | 0.00501 | 0.0776 | 11.72 | 0.0650 | 1.0000 |
| **senior + one** | senior + three or more | -0.0953 | 0.0136 | 98.24 | -6.9860 | <.0001*** |

| | | | | | | |
|---|---|---|---|---|---|---|
| **adult + two** | senior + two | -0.1003 | 0.0764 | 9.55 | -1.3140 | 0.7721 |
| **adult + two** | adult + three or more | -0.0328 | 0.0135 | 98.21 | -2.4230 | 0.1585 |
| **adult + two** | senior + three or more | -0.1331 | 0.0775 | 11.76 | -1.7170 | 0.5472 |
| **senior + two** | adult + three or more | 0.06751 | 0.0775 | 11.62 | 0.8710 | 0.9465 |
| **senior + two** | senior + three or more | -0.0328 | 0.0135 | 98.21 | -2.4230 | 0.1585 |
| **adult + three or more** | senior + three or more | -0.1003 | 0.0764 | 9.55 | -1.3140 | 0.7721 |

**Signif. codes: p <0.05 ' * ' / p<0.01 ' ** ' p<0.001 ' *** '**

**Table S10: Post hoc test (emmeans function) results of the LMM regarding Affiliative behaviors & Social Proximity, including fixed factor interaction between Max Duration and Nº Events, based on pairwise comparison of means. Results are averaged over the levels of Sex and Age Category with Tukey contrast for comparing a family of 9 estimates (adjusted p-values with Bonferroni method)**

| **Interaction Max duration : Nº events** | | estimate | SE | df | t ratio | p value |
|---|---|---|---|---|---|---|
| **short + one** | medium + one | 0.0102 | 0.0144 | 99.2 | 0.7060 | 0.9986 |
| **short + one** | long + one | -0.0691 | 0.0145 | 99.2 | -4.7630 | 0.0002*** |
| **short + one** | short + two | -0.0598 | 0.0145 | 99.2 | -4.1200 | 0.0025** |
| **short + one** | medium + two | -0.0496 | 0.0204 | 99.2 | -2.4280 | 0.2806 |
| **short + one** | long + two | -0.1289 | 0.0204 | 99.2 | -6.3320 | <.0001*** |
| **short + one** | short + three or more | -0.0754 | 0.0145 | 99.2 | -5.1970 | <.0001*** |
| **short + one** | medium + three or more | -0.0653 | 0.0204 | 99.2 | -3.1930 | 0.0471* |
| **short + one** | long + three or more | -0.1446 | 0.0204 | 99.2 | -7.1000 | <.0001*** |
| **medium + one** | long + one | -0.0793 | 0.0145 | 99.2 | -5.4630 | <.0001*** |
| **medium + one** | short + two | -0.0700 | 0.0204 | 99.2 | -3.4220 | 0.0244* |
| **medium + one** | medium + two | -0.0598 | 0.0145 | 99.2 | -4.1200 | 0.0025** |
| **medium + one** | long + two | -0.1391 | 0.0204 | 99.2 | -6.8310 | <.0001*** |
| **medium + one** | short + three or more | -0.0856 | 0.0204 | 99.2 | -4.1870 | 0.0019** |
| **medium + one** | medium + three or more | -0.0754 | 0.0145 | 99.2 | -5.1970 | <.0001*** |
| **medium + one** | long + three or more | -0.1547 | 0.0204 | 99.2 | -7.5990 | <.0001*** |
| **long + one** | short + two | 0.0093 | 0.0207 | 99.2 | 0.4510 | 1.0000 |
| **long + one** | medium + two | 0.0195 | 0.0207 | 99.2 | 0.9420 | 0.9899 |

| | | | | | | |
|---|---|---|---|---|---|---|
| **long + one** | long + two | -0.0598 | 0.0145 | 99.2 | -4.1200 | 0.0025** |
| **long + one** | short + three or more | -0.0063 | 0.0207 | 99.2 | -0.3050 | 1.0000 |
| **long + one** | medium + three or more | 0.0039 | 0.0207 | 99.2 | 0.1860 | 1.0000 |
| **long + one** | long + three or more | -0.0754 | 0.0145 | 99.2 | -5.1970 | <.0001*** |
| **short + two** | medium + two | 0.0102 | 0.0144 | 99.2 | 0.7060 | 0.9986 |
| **short + two** | long + two | -0.0691 | 0.0145 | 99.2 | -4.7630 | 0.0002*** |
| **short + two** | short + three or more | -0.0156 | 0.0144 | 99.2 | -1.0860 | 0.9750 |
| **short + two** | medium + three or more | -0.0055 | 0.0204 | 99.2 | -0.2690 | 1.0000 |
| **short + two** | long + three or more | -0.0848 | 0.0204 | 99.2 | -4.1460 | 0.0022** |
| **medium + two** | long + two | -0.0793 | 0.0145 | 99.2 | -5.4630 | <.0001*** |
| **medium + two** | short + three or more | -0.0258 | 0.0204 | 99.2 | -1.2670 | 0.9386 |
| **medium + two** | medium + three or more | -0.0156 | 0.0144 | 99.2 | -1.0860 | 0.9750 |
| **medium + two** | long + three or more | -0.0949 | 0.0204 | 99.2 | -4.6430 | 0.0004*** |
| **long + two** | short + three or more | 0.0535 | 0.0204 | 99.2 | 2.6170 | 0.1931 |
| **long + two** | medium + three or more | 0.0637 | 0.0204 | 99.2 | 3.1140 | 0.0585 |
| **long + two** | long + three or more | -0.0156 | 0.0144 | 99.2 | -1.0860 | 0.9750 |
| **short + three or more** | medium + three or more | 0.0102 | 0.0144 | 99.2 | 0.7060 | 0.9986 |
| **short + three or more** | long + three or more | -0.0691 | 0.0145 | 99.2 | -4.7630 | 0.0002*** |
| **medium + three or more** | long + three or more | -0.0793 | 0.0145 | 99.2 | -5.4630 | <.0001*** |

Signif. codes: p <0.05 ‘ * ’ / p<0.01 ‘ ** ’ p<0.001 ‘ *** ’

**Figure S1.** Plot of the residual normality distribution.

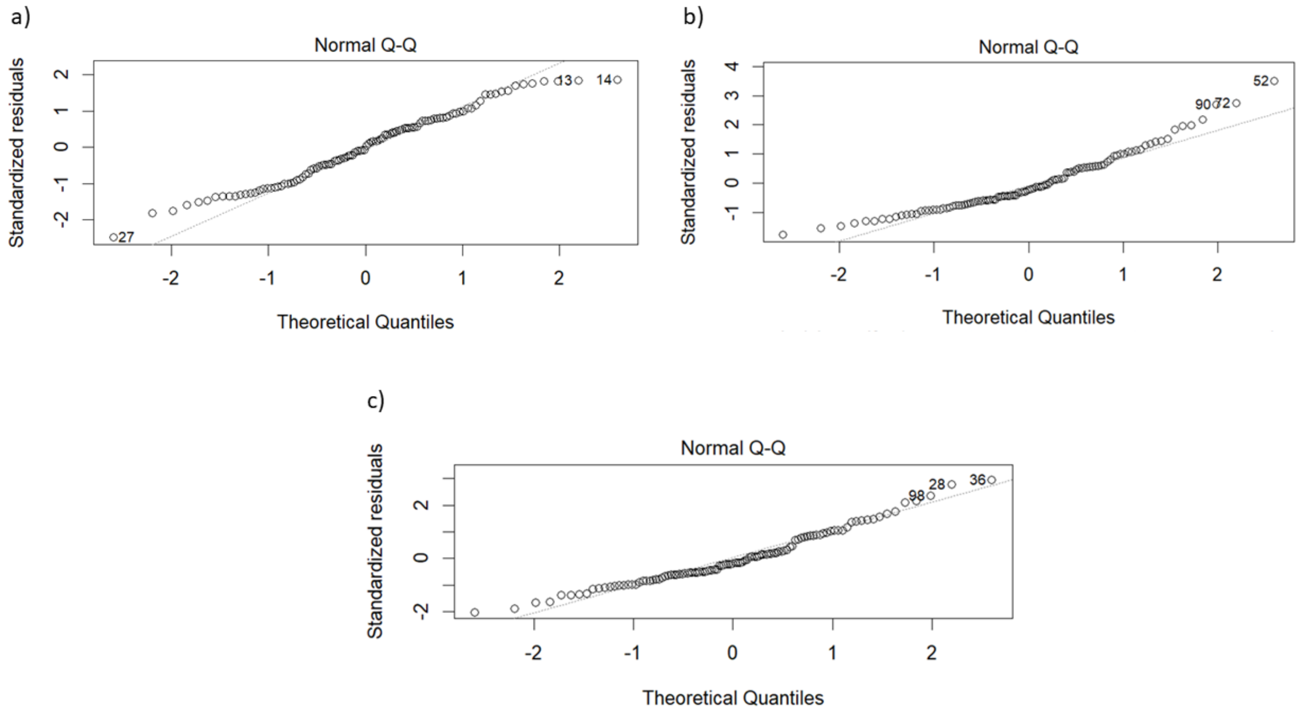


Representation of the residual normality distribution in model: a) inactivity, b) abnormal and self-directed behaviour and c) affiliative and social proximity.

**Figure S2.** Plot of the residual vs. fitted values.

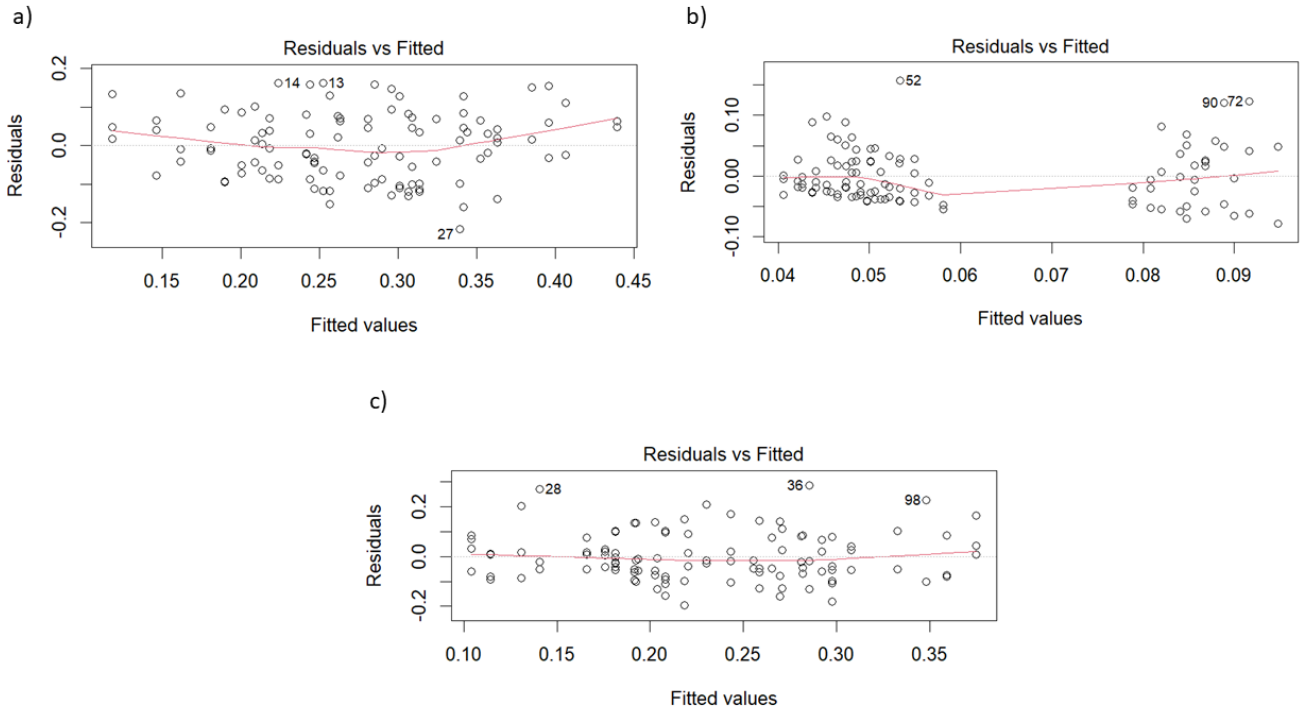


Representation of the residual vs. fitted values in model: a) inactivity, b) abnormal and self-directed behaviour and c) affiliative and social proximity.
